# Biochemical Characterization of a Novel α/β-Hydrolase/FSH from the White Shrimp *Litopenaeus vannamei*

**DOI:** 10.3390/biom9110674

**Published:** 2019-10-31

**Authors:** Karina D. Garcia-Orozco, Francisco Cinco-Moroyoqui, Lucía T. Angulo-Sanchez, Enrique Marquez-Rios, Armando Burgos-Hernandez, Jose L. Cardenas-Lopez, Carolina Gomez-Aguilar, David O. Corona-Martinez, Gloria Saab-Rincon, Rogerio R. Sotelo-Mundo

**Affiliations:** 1 Laboratorio de Estructura Biomolecular. Centro de Investigacion en Alimentacion y Desarrollo, A.C. 83304 Hermosillo, Sonora, Mexico; orozco@ciad.mx (K.D.G.-O.); xaszy17@gmail.com (L.T.A.-S.); CAROLINAAGUILAR5@hotmail.com (C.G.-A.); 2 Departamento de Investigación y Posgrado en Alimentos. Universidad de Sonora, 83000 Hermosillo, Sonora, Mexico; enrique.marquez@unison.mx (E.M.-R.); armando.burgos@unison.mx (A.B.-H.); jlcard@guayacan.uson.mx (J.L.C.-L.); 3 Departamento de Ciencias de la Salud, Universidad de Sonora, Cd. 85040 Obregon, Sonora, Mexico; david.corona@unison.mx; 4 Departamento de Ingeniería Celular & Biocatalisis, Instituto de Biotecnologia, Universidad Nacional Autonoma de Mexico, 62250 Cuernavaca, Morelos, Mexico; gsaab@ibt.unam.mx

**Keywords:** transcriptome, α/β hydrolases, esterase, lipase, immunoassays, shrimp

## Abstract

(1) Background: Lipases and esterases are important enzymes that share the α/β hydrolase fold. The activity and cellular localization are important characteristics to understand the role of such enzymes in an organism. (2) Methods: Bioinformatic and biochemical tools were used to describe a new α/β hydrolase from a *Litopenaeus vannamei* transcriptome (LvFHS for Family Serine Hydrolase). (3) Results: The enzyme was obtained by heterologous overexpression in *Escherichia coli* and showed hydrolytic activity towards short-chain lipid substrates and high affinity to long-chain lipid substrates. Anti-LvFHS antibodies were produced in rabbit that immunodetected the LvFSH enzyme in several shrimp tissues. (4) Conclusions: The protein obtained and analyzed was an α/β hydrolase with esterase and lipase-type activity towards long-chain substrates up to 12 carbons; its immunodetection in shrimp tissues suggests that it has an intracellular localization, and predicted roles in energy mobilization and signal transduction.

## 1. Introduction

A genome or a transcriptome encodes many functional proteins that can be biochemically validated by proteomic identification or by biochemical characterization of the heterologous recombinant protein. Primary amino acid sequence, and theoretical protein models provide insights into their biochemical function [1,2]. Albeit, the function of a protein cannot always be predicted only from its sequence identity. Generally, the presence of sites for ligand binding sites and functional features such as catalytic sites are essential for the prediction of its biochemical function. The catalytic sites often may be inferred from comparisons against databases of structural templates derived from active sites of multiple enzymes of a class. These methods are based on the high conservation of spatial positions of catalytic residues within functionally related enzymes, even if substrate specificities and protein folds vary significantly within the same functional class [3].

Esterases (E.C. 3.1.1) are hydrolases that catalyze the cleavage and formation of ester bonds and are represented by the canonical carboxylesterase. These enzymes are further classified into 20 subfamilies based on their substrate specificity and sequence similarity [4]. Esterases are widely distributed in nature and play a major role in the degradation of natural compounds or industrial pollutants, due to their high stereoselectivity and broad range of substrate spectrum. All esterase enzymes have a consensus sequence G-X-S-X-G [4,5].

Lipases (EC 3.1.1.3.) are enzymes that hydrolyze triacylglycerols (TAG) to free fatty acids of short or long chains [6]. They play an essential role in lipid metabolism and energy homeostasis because the fatty acids are stored as TAG, and these, in turn, are the primary endogenous source of energy. These enzymes vary widely in their primary structure, but all belong to the α/β superfamily and have a catalytic triad His-Ser-Asp within the consensus sequence G-X-S-X-G. The active site of the enzyme opens as substrate binds, by displacement of a lid loop, which exposes the water-oil interface allowing interaction of the hydrophobic substrate with the enzyme [7,8,9]. Interfacial activation is a key process unique to lipases that does not occur in esterases, requiring a regulation on the lid accessing the active site [10,11]. Besides the scientific interest in these enzymes, they have a large range of biotechnological applications [12] for regioselective synthesis and food industry [13,14,15]. In some cases, lipases are immobilized in solid supports for applications and their properties are improved [16,17,18].

The hydrolysis of emulsified TAGs (tributyrin, triolein, and olive oil) is the most important method for lipase activity determination, whereas, for esterase activity, soluble esters of short-chain fatty acids are used. Spectrophotometric assays of lipase activity generally use *p*-nitrophenyl esters of lauric or palmitic acid as substrates. Some lipases can process esterase substrates (esterase activity), and therefore substrates like *p*-nitrophenyl butyrate may be also used in lipase activity assays. Very short-chain *p*-nitrophenyl esters like *p*-nitrophenyl acetate are also used for spectrophotometric esterase assays [19].

In the post-genomic era, the experimental characterization of a gene or transcript is still required to validate the predictions made by algorithms and databases [20,21]. Many expressed transcripts are translated into polypeptides of unknown function, identified as the dark proteome [22,23]. The present work focuses on the study of a novel protein, which was discovered during the transcriptomic studies of the Pacific shrimp *Litopenaeus vannamei*. The prediction of LvFSH activity based only on the amino acid sequence is challenging. The presence of a canonical catalytic Asp/His/Ser triad and the theoretical modeling suggests that it belongs to the α/β hydrolase fold family. Further experimental validation showed that LvFSH is a functional intracellular esterase with a possible signaling function.

## 2. Materials and Methods

### 2.1. Amino Acid Sequence of α/β Hydrolase (LvFHS)

The amino acid sequence was deduced from a transcript identified in a transcriptome of white shrimp *Litopenaeus vannamei* reported by Ghaffari et al. (2014) [20] and later identified in the shrimp genome as GenBank XP_027218885.1.

### 2.2. LvFHS Sequence Features

Tools such as Pfam (Protein Families Database of Alignments and HMM http://pfam.xfam.org [24], InterPro (protein sequence analysis and classification http://www.ebi.ac.uk/interpro), PRINTS (http://umber.sbs.man.ac.uk/dbbrowser) BLAST, PROSITE (http://ca.expasy.org/cgi-bin/prosite), Yuves (http://prodes.toulouse.inra.fr/prodom/current/html/home.php), SMART (Simple Modular Architecture Research Tool http://smart.embl-heidelberg.de/), and ELM (Eukaryotic Linear Motif http://elm.eu.org), were used for the identification of functional domains in the target amino acid sequence of this study. Putative sites for protein-protein interactions were identified using the STRING algorithm (http://string-db.org), and in order to identify a possible signal peptide and post-translational modifications, the sequence was analyzed using the portal SignalP 5.0 (http://www.cbs.dtu.dk/services/SignalP) and also NetPhos 3.1 (http://www.cbs.dtu.dk/services/NetPhos), while glycosylation prediction was made on the server YinOYang 1.2 (http://www.cbs.dtu.dk/services/YinOYang).

The LvFSH amino acid sequence was analyzed to propose a possible cellular localization using the PORT WWW Server site (Prediction of Protein Sorting Signals and Localization Sites in Amino Acids Sequences https://psort.hgc.jp), WoLFPSORT Prediction, PSORT II Prediction, and Prediction iPSORT. In addition, we employed TargetP 1.1 Server (http://www.cbs.dtu.dk/services/TargetP) and CELLO v.2.5 (subcellular Localization predictor http://cello.life.nctu.edu.tw) and BaCelLo (Balanced Subcellular Localization Predictor (http://gpcr2.biocomp.unibo.it/bacello/index.htm). TargetP 1.1 predicts the eukaryotic protein subcellular location. The assignment of location is based on the prediction of any N-terminal pre-sequences such as peptide transit (cTP) to chloroplast, mitochondrial orientation (mTP) peptide, or signal peptide of the secretory pathway (SP). For sequences predicted to contain an N-terminal peptide sequence, potential spin-off sites can also be predicted.

### 2.3. Protein Structure Modeling

A three-dimensional structural model of LvFSH was obtained using the Phyre2 algorithm (http://www.sbg.bio.ic.ac.uk/phyre2/html/page.cgi?id=index) [25]. The quality of the model obtained in Phyre2.0 was evaluated with the ProQ2 tool within the same Phyre2 platform, in addition to the ProSA server (https://prosa.services.came.sbg.ac.at/prosa.php). The structural figures were created using PyMol [26]. The molecular volume of the *p*-NPA and *p*-NPL were calculated using the crystallography software Olex2 [27]. The substrate was optimized in its 3D structure using the Avogadro software [28], applying the molecular mechanics calculations.

### 2.4. Recombinant Expression and Purification of LvFSH

The LvFSH amino acid sequence (GenBank XP_027218885.1) was used to construct a synthetic gene for recombinant expression in *E. coli*, with a 10-His residue tag at the N-terminus. The construct was comprised of the LvFSH coding region with *E. coli* optimized codons, under the control of the T7-promoter on the pJexpress414 (DNA2.0) expression vector. The plasmid was used to transform a sodium chloride-inducible *E. coli* strain (BL21DE3-SI), that requires both NaCl and IPTG to induce recombinant protein expression. All chemicals and reagents were from Sigma-Aldrich unless mentioned.

From a single transformed colony, a 25 mL LB broth (100 μg/mL ampicillin and 30 μg/mL chloramphenicol) starting culture was made and used to inoculate 1 L LB broth with ampicillin, with stirring in an orbital shaker at 225 RPM and 37 °C. LvFSH expression was induced when the culture reached an optical density of 0.6, by the addition of IPTG to a final concentration of 1 mM, and NaCl to 0.3 M. The centrifuged bacterial pellet was collected by centrifugation and stored at −80 °C.

A total of 1 g of the bacterial pellet was mixed with 5 mL of lysis buffer containing 20 mM Tris-HCl pH 7.4, 1 mM DTT, 0.5 mM PMSF, 5 mM benzamidine, 0.5 M NaCl, and 0.1 mg/mL hen egg-white lysozyme. The bacterial suspension was sonicated on an ice bath with 10 pulses of 60 s each, and then it was centrifuged at 35,000× *g* for 30 min at 4 °C. Then, 0.7% streptomycin was added to remove DNA, and clarified by centrifugation at 35,000× *g* for 25 min.

The recombinant protein, LvFSH, was purified by Ni^+2^ affinity chromatography (IMAC) using an ÄKTA chromatographer (GE Healthcare). The clarified protein extract was dialyzed with buffer A containing 20 mM Tris-HCl pH 7.4, 500 mM NaCl, and was loaded in a 5 mL His-Trap column previously equilibrated with buffer A. The column was washed with buffer A to remove non-specific protein. Elution of the His-tagged protein was performed with a gradient from 0 to 500 mM imidazole in buffer A, and 3 mL fractions were collected.

A second purification step was required. The fraction containing LvFSH was equilibrated with a buffer containing 25 mM sodium phosphate pH 7.4 and 3 M NaCl and loaded into a 5 mL hydrophobic interaction column. A gradient of 25 mM sodium phosphate pH 7.4 was used to elute the protein. The LvFSH protein was quantified using the bicinchoninic acid method (Pierce BCA Protein Assay Kit, Thermo Scientific). The samples were read at 595 nm in a microplate reader (iMark Microplate Reader, Bio-Rad), and the protein concentration was calculated using a bovine serum albumin standard curve in mg/mL.

The purification process was monitored by polyacrylamide gel electrophoresis in the presence of sodium dodecyl sulfate (SDS-PAGE) and under reducing conditions, using 1 mm, 15% polyacrylamide gels. The samples were prepared by mixing with 2X loading buffer (240 mM Tris pH 6.8, 10% SDS, 10% β-mercaptoethanol, 0.04% bromophenol blue, 20% glycerol), and were heated for 10 min at 96 °C before loading onto the gel. Electrophoresis was carried out at constant power 15 mA (PowerPac HV, Bio-Rad) into the gel (Mini-PROTEAN B Cell, Bio-Rad), using Tris-glycine buffer (0.025 M Tris base, 0.192 M glycine, 0.1% SDS) buffer. Unstained SDS-PAGE, broad range, Bio-Rad molecular weight standards were used. The protein was visualized by fluorescence, by adding 0.5% (*v*/*v*) 2,2,2-trichloroethanol (TCE) to the polyacrylamide mix [29].

### 2.5. Secondary Structure Analysis by Circular Dichroism (CD)

Circular dichroism (CD) spectra were acquired in a spectrophotopolarimeter (Jasco J-810) using a 0.1 cm path-length cell in the far-UV range (190–240 nm). The CD spectra were recorded at 25 °C, every 0.5 nm with a 4 s average time/point and a 1 nm band-pass with a scan speed of 100 nm/min. Protein concentration was 1.74 mg/mL. Spectra were corrected for the blank 25 mM phosphate buffer, pH 7.4, and smoothed. Secondary structure content was determined by analysis of the delta epsilon units (∆e) with the SELCON algorithm available on the DICHROWEB server (http://www.cryst.bbk.ac.uk/cdweb/html/) at Birkbeck College, UK [30].

### 2.6. Enzymatic Assays

Hydrolase enzymatic activity was measured by a modified spectrophotometric method that monitors the rate of hydrolysis of *p*-nitrophenyl esters [31]. The enzyme solution was assayed at 37 °C with 50 mM Tris-HCl, pH 8.0, containing 0.2% (*v*/*v*) Triton X-100 and 50 mM NaCl. For esterase activity, the substrate chosen was *p*-nitrophenyl acetate (*p*-NPA), whereas, for the lipase assay, *p*-nitrophenyl laurate (*p*-NPL) was chosen. Both compounds were dissolved in ethanol and used to start the reaction. Then, 2 mL reactions were set and read on a diode-array UV spectrophotometer (Agilent 8453) equipped with a 1 cm path-length quartz multi-cell. Lipase activity was measured at 410 nm against a blank containing no enzyme for self-monitoring spontaneous hydrolysis of the substrate. One unit of lipase activity was defined as the amount of enzyme required to release 1 μmol of *p*-nitrophenol per min under the conditions of the assay. This was calculated using an extinction coefficient of ε_410 nm_ = 12,750 cm^2^/mmol. The effect of the substrate concentration on the reaction rate was determined, and the Michaelis–Menten Km and Vmax were calculated for both substrates assayed.

Dihydrofolate reductase enzymatic activity was measured using a spectrophotometric NADHP-oxidation assay [32]. A set of reagents containing dihydrofolic acid and NADPH stock solutions were used. Absorbance was measured at 340 nm in an UV-visible spectrophotometer (Cary 50, Varian, Palo Alto, CA, USA) in a 1 cm path-length quartz cell. In all enzymatic assays, recombinant LvFSH was used at 60 μg protein/mL of final reaction volume in the quartz cell.

### 2.7. Production and Purification of Anti-LvFSH Polyclonal Antibodies

A three-month-old male rabbit was acclimated to captivity at 25 °C, and it was fed daily with pet food (Purina ^MR^). A total of 1 mL of the recombinant LvFSH at 2 mg/mL was mixed (1:1, *v*/*v*) with complete Freund’s Adjuvant (Sigma-Aldrich, Toluca, Mexico) to obtain 2 mL at a final concentration of 1 mg/mL. The rabbit was immunized by subcutaneous injection, and after 28 days, a second booster was applied as described above but using incomplete Freund’s adjuvant. One week after the second immunization, the rabbit was bled from the ear vein. The blood was set for 2 h to separate the serum, then it was centrifuged at 15,000 rpm, and stored at 4 °C until the purification of antibodies [33].

The purification of rabbit IgG antibodies was carried out by affinity chromatography. A protein A column [34] previously equilibrated with a buffer containing 0.1 M Tris-HCl, 1 M NaCl pH 8.6, was used. The serum was diluted in equal parts (*v*/*v*) and equilibrated for 10 min with dilution buffer (0.2 M Tris-HCl, 2 M NaCl pH 8.6), then was applied to the column at a flow of 1.5 mL/min. The non-bound protein was washed with the same buffer. The IgG′s attached to the column (LvFSH antibodies) were eluted with 0.1 M sodium citrate, pH 3. Fractions were collected in cold neutralization buffer (K_2_HPO_4_ 0.2 M pH 9, 4 °C) in equal parts (*v*/*v*) to keep a neutral pH. Using an ultrafiltration cell (Amicon), the purified anti-LvFSH was diafiltrated in a cold bath (4 °C) with two volumes of saline solution and concentrated using a 10 kDa membrane. Finally, antibodies were stored at 4 °C in saline solution with 10 mM sodium azide.

The antibodies were tittered using an immune dot-blot assay. Strips (1.5 × 10 cm) of 2 μm PVDF were used. Dilutions (1:10, 1:100, 1:1000, 1:10,000, 1:100,000, 1:1,000,000) in Tris buffer saline (TBS; 20 mM Tris-HCl, 500 mM NaCl pH 7.5) of the LvFSH protein (antigen) were made. Then, 2 μL of each dilution were adsorbed on the membranes. The membranes were blocked overnight with TBS, 5% Blotting Grade Blocker (Bio-Rad), and 2% Tween 20 on a rocking platform. A total of 2 μL of TBS was applied to each strip as a negative control, and the experiment was conducted in triplicate.

Three dilutions of the anti-LvFSH immunoglobulins were made (1:500, 1:1,000, 1:1,500), using an incubation buffer (20 mM Tris-HCl, 500 NaCl mM, 0.05% Tween 20 pH 7.5). Strips containing LvFSH after blocking were incubated in the three dilutions for 1 h. The strips were washed three times for 5 min with TBST (20 mM Tris-HCl, 500 mM NaCl, 0.05% Tween 20 pH 7.5). After washing, the membranes were incubated for 1 h with a 1:5000 dilution of secondary antibody conjugated with alkaline phosphatase (Goat anti-rabbit-AP; Bio-Rad). Next, three washes for 5 min with washing buffer were undertaken. Finally, the membranes were washed 1 min with distilled water. The color was developed by the addition of the AP substrate in buffer 100 mM Tris-HCl, 0.5 mM MgCl_2_ pH 9. The enzymatic reaction was stopped by washing the membrane with distilled water for 10 min and drying the membranes at room temperature. Three controls were carried out, without any antibody and excluding the first and second antibodies.

### 2.8. Detection of LvFSH on Shrimp Tissues

Ten adult white shrimp (*Litopenaeus vannamei*) of 6 g (Laboratorio de Fisiología de Invertebrados Marinos at the Centro de Investigación en Alimentación y Desarrollo; CIAD) were dissected to obtain samples of muscle, pleopods, gills, intestine, posterior intestine, stomach, and hepatopancreas. The tissues were stored at −80 °C until their use for protein extraction, according to Rivera-Pérez et al., 2011 [35]. Protein was extracted on an ice bath by adding buffer 50 mM Tris-HCl pH 8, 150 mM NaCl, 0.5 mM PMSF to tissues at a ratio of 1:1 (*w*/*v*), and sonicated by four pulses of 10 s. The homogenate was centrifuged at 13,000× *g* at 4 °C. The clarified and pellet were separated, and the protein concentration of clarified extracts was determined by the acid bicinchoninic method. Samples were analyzed by SDS-PAGE as described above.

### 2.9. Enzyme-Linked Immunosorbent Assay (ELISA)

The assays were performed on ELISA 96-well microplates at room temperature. The protein extracts from the different tissues were diluted (1:1) with carbonate buffer (15 mM Na_2_CO_3_, 35 mM NaHCO_2_, pH 9.6). A total of 100 μL of each sample was loaded and incubated overnight on an orbital shaker. The excess liquid was discarded, and the plate was washed with 200 μL of carbonate buffer for 3 min and dried. Then, 200 μL of blocking buffer (100 mM Tris-HCl, 0.05% Tween 20, 0.05% phenol red, 5 mM sodium azide, 1% Blotting Grade Blocker Bio-Rad pH 7.3) was added to each well and incubated for 20 min with agitation. Four washes were performed for 3 min by adding 200 μL wash/dilution buffer (100 mM Tris-HCl, 0.05% Tween 20, 0.05% phenol red, 5 mM sodium azide pH 7.3). A total of 100 μL of dilution (1:5000) of the anti-LvFSH antibody was added and incubated on agitation for 1 h. Subsequently, four washes for 3 min, with 200 μL wash/dilution buffer were performed. Then, 100 μL of 1:2000 diluted second antibody was added, and the plate was incubated for 20 min with agitation. Four washes for 3 min with 200 μL wash/dilution buffer were performed. Next, a wash for 3 min with 200 μL of 50 mM sodium phosphate pH 7.4 buffer was performed. The color was developed by the addition of the AP substrate (Alkaline Phosphatase Substrate Kit, BioRad). Finally, the reaction was stopped by the addition of 150 μL of 0.4 mM NaOH. The reaction was read at 415 nm on a microplate reader. The samples were analyzed in duplicate, and the concentration of LvFSH in the protein extract was calculated using a standard LvFSH protein curve previously carried out. The concentration of LvFSH was expressed in mg per g of tissue. A comparison analysis of Tuckey′s means (*p* < 0.05) and a one-way analysis of variance (ANOVA) were performed.

## 3. Results

### 3.1. Sequence Analysis of LvFSH

The BLASTp of the deduced amino acid sequence of *LvFSH* identified in a transcriptome of the white shrimp *Litopenaeus vannamei* had a high identity with members of the superfamily of α/β hydrolases, more specifically, with serine hydrolases (FSH) and serine esterases, which include the carboxylesterases, thioesterases, cholinesterases, and lipases. Therefore, the BLASTp results were not conclusive to predict for a specific enzymatic activity of LvFSH.

On the other hand, the sequence of this study was annotated in the recently reported genome of the shrimp *Litopenaeus vannamei* as esterase *OVCA2*-like (GenBank XP_027218885.1) [21]. *OVCA2* has been reported in humans as a key tumor suppressor in different types of cancer. Upon analysis of the *OVCA2* amino acid sequence, it is evident that the gene product belongs to the α/β hydrolases superfamily due to conserved serine hydrolases (FSH) domains. However, there are no reports of enzymatic activity on the human OVCA2 protein. Sequence analysis of the shrimp gene using FASTA and PSI-FASTA (https://www.ebi.ac.uk/Tools/services/web/) yielded similar inconclusive results to those mentioned above. Quevillon et al. (2005) carried out a combined analysis of experimental and computational proteomics data for the detection of active serine hydrolase activity in yeast [3]. They reported an FSH1 protein that, together with two other serine hydrolases (FSH2/FSH3), is a member of a new eukaryotic FSH family. This group includes two proteins: OVCA2 (associated with cancer in humans) and DHFR (a dihydrofolate reductase from *Schizosaccharomyces pombe*). No DHFR activity was observed in recombinant LvFSH using dihydrofolate as a substrate.

**Figure 1:**
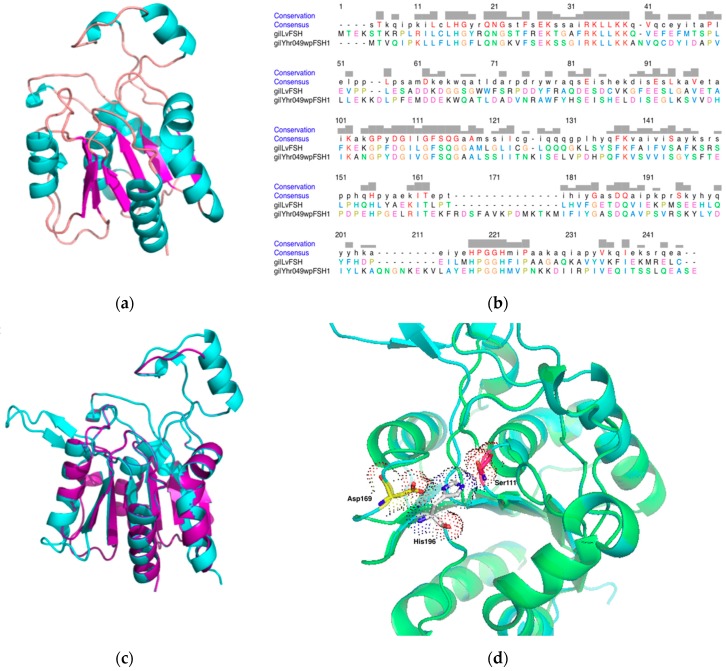
Comparation and analysis of LvFSH model obtain by Phyre2.0 (**a**) Molecular modeling of the structure of *Litopenaeus vannamei* LvFSH. Ribbon representation helices, strands, and loops are colored (α-helices cyan, β-sheet magenta, and loops orange, respectively); (**b**) alignment of LvFSH from shrimp (this study) and Yhr049wp (*Saccharomyces cerevisiae*); (**c**) structural alignment of model LvFSH (green) with FSH1/YHR049W from *S. cerevisiae* structure (PDB code 1YCD) (cyan); (**d**) putative catalytic triad Ser111 (red), Asp169 (yellow), and His196 (green).

Esterase enzymes act on carboxylic esters. The catalytic system involves three amino acid residues called the catalytic triad, a serine (Ser), an acidic residue like glutamate (Glu) or aspartate (Asp), and a histidine (His) residue. These catalytic residues are responsible for the nucleophilic attack at the carbon of the carbonyl ester linkage. These sequences are predicted to have the α/β superfamily hydrolase fold (Figure 1a). In comparison to other members of the superfamily α/β hydrolase, esterases of the *p*-nitro benzyl type and acetylcholine type have on the carboxylate of the active site a Glu residue instead of Asp. Figure 1b shows the alignment of the sequence in this study (LvFSH) with other members of the FSH α/β hydrolase family from different species. Sequence conservation is evident, and it suggests that structural and functional elements and functions are also shared by these proteins.

### 3.2. Comparison of LvFSH Domains

Multiple sequence alignment of LvFSH with the InterPro and Pfam tools to search for common domains with known proteins showed high identity (residues 6–217) with the α/β hydrolase superfamily. A characteristic domain of serine hydrolases (FSH) was also identified from residues 8 to 202 of LvFHS. These two regions were identified as homologous of protein superfamily 1.5, according to database analysis and function (HMM library and genome assignments server; http://supfam.org/suprfamily/index.html) and CATH (Class, Architecture, Topology, Homology; http://www.cathdb.info/).

The α/β hydrolase fold consists of a central parallel β-sheet sandwiched between major α-helices. Esterases, carboxylesterases, thioesterases, lipases, and the current FSH. Other uncharacterized proteins like FSH1/YHR049wp, FSH2/YMR222cp, FSH3/YOR280cp of *Saccharomyces cerevisiae*, dihydrofolate reductase DYR_SCHPO (SP36591) from *Schizosaccharomyces pombe* and human tumor suppressor OVCA2 also show this characteristic fold [3].

The folding of α/β hydrolase [7] is common to several hydrolytic enzymes of different phylogenetic origin and catalytic roles. It is accepted that these enzymes have evolved from a common ancestor, preserving the layout of the catalytic residues. In most of the members of this superfamily the β-sheets are parallel in the core of the enzyme, but in some cases, an antiparallel orientation occurs by the inversion of the first chains. All have a catalytic triad, whose residues are located on loops, one of them named “elbow nucleophile”. Some other members lack one or all of the catalytic residues. Therefore, these members are inactive, but other residues are involved in recognition of the substrate surface. The ESTHER database contains published information related to the sequences of genes and proteins in this superfamily [36]. *Pseudomonas putida* esterase (EST) is a member of the superfamily of enzymes α/β hydrolase.

When SMART was used to analyze the LvFSH sequence, in addition to the FSH domains, structural homology was also found with the d1auoa protein from the SCOP database (residues 3–214, value E 1e^−8^) and with an experimental crystallographic structure 1ycd PDB (residues 11–199, Value E 8e^−14^). With lower significance (high E-value) were the DLH domain from the Pfam database (residues 71–202) characteristic of the family of dienelactone hydrolases [37,38] and YccV, a DNA-binding protein from the SMART database (residues 107–192) [39]. The search with ProDom and PRINTS identified the same domains. However, it showed a statistically significant alignment with an uncharacterized DHFR type protein (DYR_SCHPO P36591) from *Schizosaccharomyces pombe* mentioned above [40].

Functional motifs or possible sites of posttranslational modification were detected by ELM analysis. ELM (Eukaryotic Linear Motif ELM resource) is a computational biology resource that annotates and identifies eukaryotic linear motifs (ELMs), by providing a repository of annotated motif data, and prediction tools. ELMs, or short linear motifs (SLiMs), are compact amino acid sequences within proteins that are predicted to provide protein interaction sites. These motifs are commonly comprised of intrinsically disordered regions of the proteome, and provide a wide range of functionality to proteins [41,42]. ELMs play crucial roles in cell regulation, and SLiM mimics are often used by pathogens to manipulate their hosts′ cellular machinery [41,43]. In the analysis of LvFHS, 16 potential functional sites were found in the sequence. The ELM motifs listed in Table 1 are those located in the solvent-accessible surface from the LvFSH model obtained by Phyre2.

SLiMs maybe overpredicted, however, precision can be improved by using additional filters based on contextual information, including taxonomy, cellular compartment, evolutionary conservation, and structural features. It has to be considered that the tertiary structure of the protein is not yet known. Therefore, some of the motifs detected by the web server might be positioned in a globular region distant from the surface, then their role in vivo would have to be reevaluated [44].

Using PROSITE and Predict Protein tools, a potential glycosylation site (*N*-glycosylation) was identified, five phosphorylation sites for protein kinase C (positions 2–4, 5–7, 24–26, 132–134); four sites for casein kinase II phosphorylation (24–27, 47–50, 56–59, 69–72) and four sites of N-myristoylation (92–97 114–119 118–123, 202–207), were identified. It will be important to review the location of these sites in a theoretical and/or experimental structure.

### 3.3. Protein-Protein Interactions

Assuming the homology of LvFSH with the protein FSH3 of *Saccharomyces cerevisiae*, the possible interaction with other protein sites was investigated with the STRING tool (http://string-db.org). The most relevant result was a possible interaction with the domain LIDH hydrolase (Pfam) present in the protein LDH1 (lipid droplet hydrolase). LDH1 is a serine hydrolase with esterase and lipase activity towards triacylglycerols. It has been proposed that LDH1 participates in lipid homeostasis, regulating the levels of phospholipids and non-polar lipids and that are required for the mobilization of lipids stored in small droplets where the enzyme is localized [45]. Thus, the possible interaction predicted may indicate a physical colocalization of LvFSH and LDH1 that would lead to a more efficient fat mobilization at the lipid droplet.

### 3.4. Signal Peptide and Post-Translational Modifications

SignalP 5.0 (http://www.cbs.dtu.dk/services) did not reported the presence of a signal peptide in the query sequence (LvFSH). NetPhos 3.0 predicted nine serine residues (positions 5, 23, 47, 56, 64, 90, 130, 132, and 177) five of threonine (2, 6, 24, 96, and 161) and tyrosine 174 as possible phosphorylation sites. Serine 140 was found susceptible to N-acetyl glycosylation. Threonine 2 and serine 5 were predicted to be *O*-GlcNAcylated, as well as phosphorylated, indicating that such sites can be reversibly and dynamically modified by *O*-GlcNAc or phosphate groups at different times in the cell [46]. These results suggest that LvFSH is susceptible to post-translational regulation via phosphorylation.

### 3.5. LvFSH Subcellular Localization

The prediction of LvFSH localization using PSORT II,CELLO and BaCelLo tools found the mitochondria as the probable location the LvFSH protein. The iPSORT Prediction and TargetP 1.1 Server tools detected an *N*-terminal mitochondrial orientation peptide in LvFSH. A possible role within the mitochondria for a hydrolase remains to be addressed, potentially during lipid mobilization stages such as starvation.

### 3.6. Theoretical Modeling of LvFSH

Figure 1a shows the structure obtained from modeling performed in Phyre2. This model is based on a crystal structure (PDB 1ycd), which was obtained from a 27 kDa protein with unknown function encoded by the Yhr049w gene called *FSH1*. Structural genomics aims to obtain the structure of proteins predicted in a genome, especially if the sequence is novel and the structural prediction is not obvious. In this case the structure led to a novel yeast serine hydrolases [47,48]. ScFSH1 has around 23% identity with LvFSH (Figure 1b), although Phyre2 was able to produce alternative models with protein structures (PDB) with a lower degree of identity, but all belong to the α/β hydrolase superfamily.

The characteristic α/β hydrolases fold was identified as a likely scaffold for holding the catalytic site (Figure 1c). The protein model has a six-stranded central parallel β-sheet surrounded by six major alpha-helices in order β2β1β3β4β5β6. A full helix (α2) and two short helical turns connect the β2 sheet and the α3 helix to form a cap-like extension that covers the catalytic site. The theoretical model obtained does not contain the classical arrangement of antiparallel β sheets (β5β6) that conforms the lid that closes the channel to the active site [49,50]. However, since the enzyme is active, this finding will require the experimental determination of the crystal structure to know the actual fold in the vicinity of the active site

The α/β-hydrolase canonical fold is comprised of an eight-stranded mostly parallel β-sheet, and Yhr049w and FSH1 lacked the first two β1 and β2 strands. This is the case of other hydrolases, such as the bacterial lipase [51], cutinase [52], and *Streptomyces scabies* esterase [53].

The theoretical model of LvFSH has the canonical fold of the α/β hydrolases with a catalytic triad consisting of a nucleophile, a histidine, and an acidic residue (Figure 1d). The nucleophile and the acid may change between protein families. This residue can be a cysteine, serine (usually), or aspartate residue as in the case of haloalkane dehalogenases. The acid can be aspartic or glutamic acid. In the sequence of LvFSH, we propose that the catalytic triad residues are Ser111, Asp169, and His196 (Figure 1d) [3,49,54]. The residues of the catalytic triad are situated at the C terminal end. The nucleophile Ser111 is situated at the end of strand β3, in an acute vertex with unfavorable Ramachandran angles [7,50]; Asp169 located at the end of the strand β5 in the loop that joins it with the helix α5, finally the His96 located at the end of strand β6 in the loop that joins it to helix α6. The model obtained lacks a wall to form a channel in the catalytic site (sheet (β7β8), this remains as an open site, which could lead to the interaction of larger substrates such as lipid molecules.

Aligning the model built in Phyre2 for the sequence in PDBeFold (http://www.ebi.ac.uk/msd-srv/ssm/) showed the loop around the active site is conserved with the carboxylesterases and the acyl thioesterases. It has been observed that although the identity between the sequences of all these enzymes can be small (16%), the Phyre2 algorithm predicts a very similar molecular architecture.

**Figure 2:**
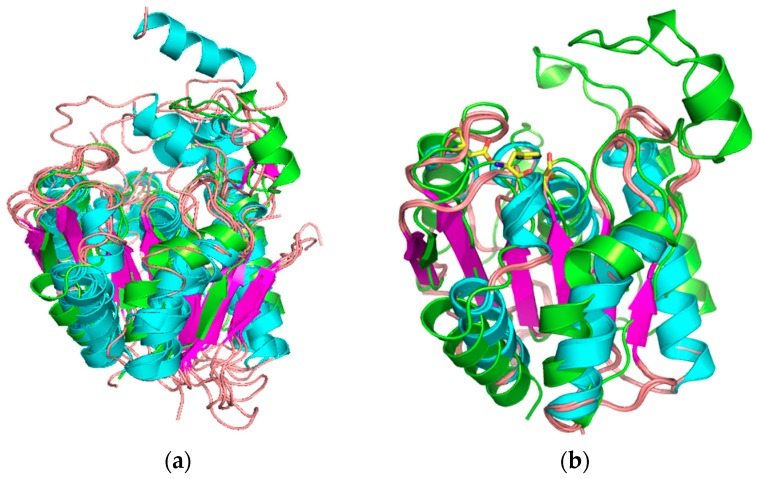
Structural comparison of model LvFSH. α/β-hydrolase models were obtained from the MSD server (http://www.ebi.ac.uk/msd-srv/ssm/); (**a**) esterase structures (root mean square deviation (RMSD) 2.24 Å, Q-score 0.2836 for 137 Cα); (**b**) lipase structures (RMSD 1.127 Å, Q-score 0.4678 for 149 Cα). The LvFSH model is shown in green.

The comparison of the model obtained versus the PDB database structures using the MSD server (http://www.ebi.ac.uk/msd-srv/ssm/) revealed that LvFSH was similar to 35 different structures with an overall root mean square deviation (RMSD) of 2.05 Å (six carboxylesterases, esterases, and with thioesterases with an RMSD of 2.24 Å; Figure 2a), and with 20 phospholipases and lipases with an RMSD of 1.13 Å (Figure 2b), and nine uncharacterized α/β hydrolases (RMSD of 2.46 Å). The model obtained and compared with lipases showed an extra strand around the active site. This extended groove loop was reported for carboxylesterase and thioesterase enzymes as well [51].

**Figure 3:**
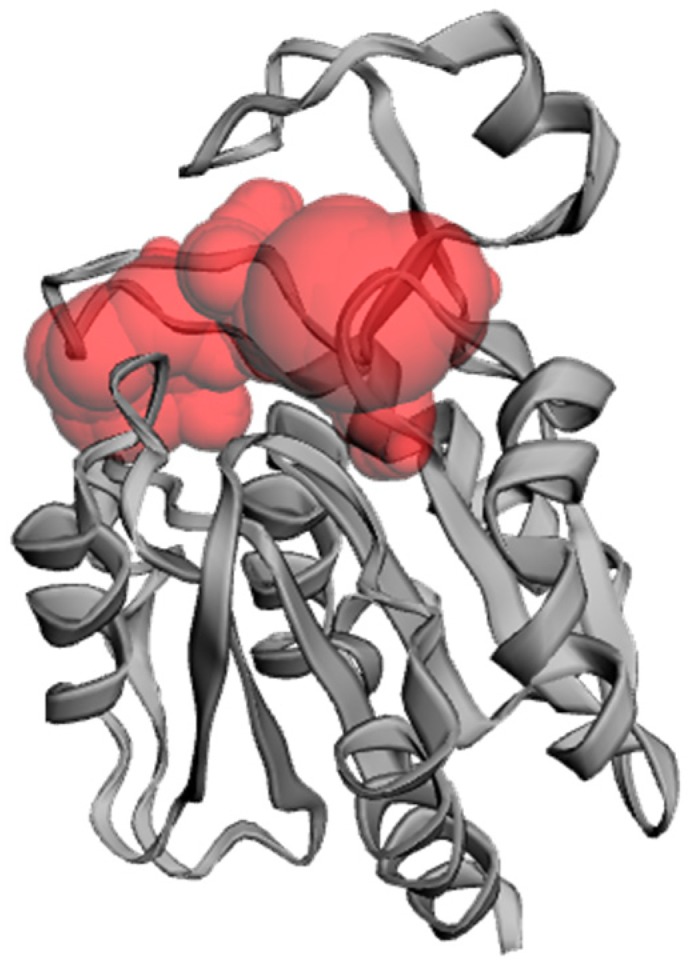
Substrate binding pocket in the LvFSH model. A binding pocket was predicted, and it is shown in red with a volume of 309 Å^3^.

A potential substrate binding pocket within the model was found using CASTp (Computed Atlas of Surface Topography of proteins, http://sts.bioe.uic.edu/castp/calculation.html; [55]) (Figure 3). The binding pocket is located in the vicinity of residues Ser111 and His196, reinforcing the hypothesis that these are catalytic residues. Note that the substrate-binding pocket is well accessible and able to accommodate a large variety of substrates, evidenced by the large variety of reactions that α/β serine hydrolases catalyze. However, the volume of the cavity (309 Å^3^) is comparable to the main binding pocket in an OVCA2 model [44] indicating the enzyme could be a carboxylesterase.

We calculated the volume of the substrates evaluated experimentally in this work. The molecular volumes of *p*-NPA and *p*-NPL were 146 and 298 Å^3^, respectively. Both substrates fit in the 309 Å cavity of the active site, and this allows us to predict that substrates with a chain longer than 12 carbons would bind with low affinity.

**Figure 4:**
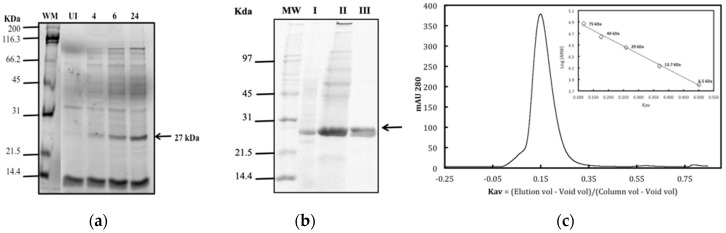
SDS-PAGE of LvFSH purified by two chromatographic methods. (**a**) MW: molecular weight marker. UI, not induced, and 4, 6, 24 h post-induction with 1 mM IPTG. 0.3 M NaCl; (**b**) SDS-PAGE purification of LvFSH by chromatography methods chromatography, MW Molecular Weight Marker Broad Range (Bio-Rad). I, crude extract; II elute with 23% of imidazole 500 mM Ni^+2^ affinity chromatography (IMAC) and loaded to HIC; III, pure LvFSH obtained after hydrophobicity chromatography; (**c**) molecular exclusion chromatography. A calibration curve was constructed using the molecular weight standards; conalbumin (75 kDa), ovalbumin (44 kDa), carbonic anhydrase (29 kDa), A ribonuclease (13.7 KDa), and aprotinin (6.5 kDa) in the same buffer. Inset shows the log of MW of standard proteins vs. elution volume.

Soluble recombinant protein from *Litopenaeus vannamei* LvFSH was obtained in *E. coli* BL21 bacteria after 24 h post-induction with 1 mM IPTG and 300 mM NaCl (Figure 4a). The recombinant LvFSH was purified to homogeneity using nickel affinity chromatography (IMAC) and hydrophobic interaction. The purified preparation was homogenous as judged by the presence of a single dominant protein band of approximately 27 kDa on SDS-PAGE (Figure 4b, lane III). The molecular weight of the native enzyme was calculated to be approximately 58 kDa by gel filtration chromatography, indicating that in solution, LvFSH is probably a dimer (Figure 4c).

### 3.7. Secondary Structure Analysis of LvFSH

**Figure 5:**
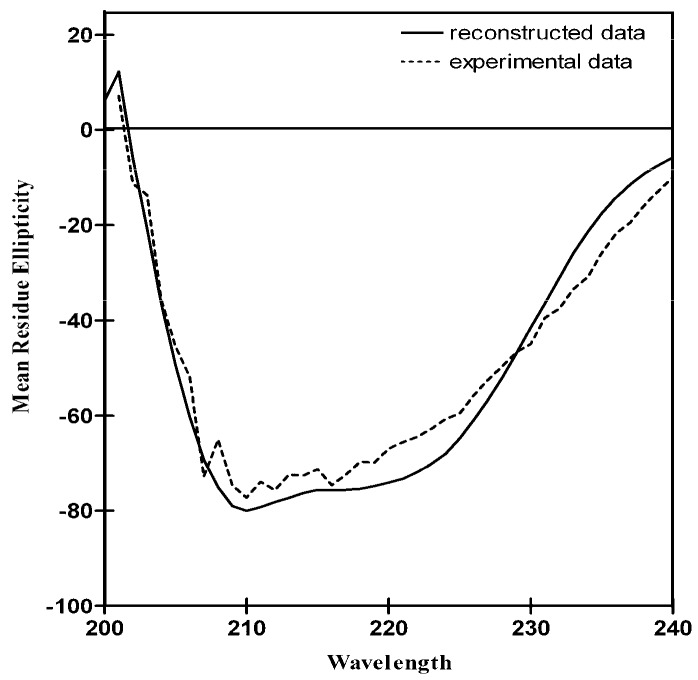
Far-UV circular dichroism (CD) spectrum of LvFSH represented as mean residue ellipticities (degree cm^2^ dmol^−1^ residue^−1^ × 10^3^). The experimental data (discontinuous line) and the reconstructed spectrum (continuous line) as calculated by K2D algorithm [56].

Analysis of the LvFSH mean residue ellipticities led to a calculated secondary structure comprised of 31% α-helix, 18% β-sheets, and 51% unordered structure (Figure 5). This experimental result is comparable to the secondary structure assignment from the Phyre2 model. Therefore, there is agreement between the theoretical structural assignment and an experimental estimation of the secondary structure content method.

### 3.8. LvFSH Enzymatic Activity

Enzymatic activity was tested using *p*-NPA and *p*-NPL as substrates. Specific activities for LvFSH were 10.99 U per mg protein for the former and 0.77 U/mg for the latter substrate. The characteristic esterase enzymes are only active with short-chain fatty acid esters (no more than six carbons) using *p*-NPA as substrate. However, LvFSH was active on short- and long-chain insoluble fatty acid esters (*p*-NPL, 12 carbon chain).

**Figure 6:**
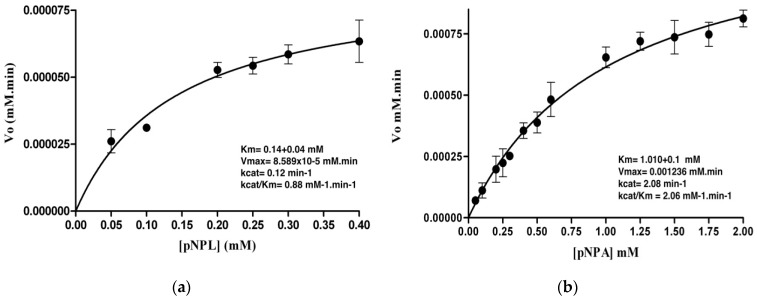
Effect of substrate concentration [S] on the enzymatic rate (V_o_) of LvFSH, using *p*-nitrophenyl esters. (**a**) A 0.05–0.2 mM concentration range was assayed for *p*-nitrophenyl laureate (*p*-NPL, 12-carbon chain); (**b**) a 0.05–2 mM concentration range was assayed for *p*-nitrophenyl acetate (*p*-NPA, 1-carbon chain). Kinetic parameters were calculated. Three replicates were assayed.

The effect of substrate concentration (range 0.05–0.2 mM for *p*-NPL and 0.05–2 mM for *p*-NPA) on the hydrolysis rate is shown in Figure 6. For both substrates, the reaction rate increased with some saturation at the highest concentration for *p*-NPL (Figure 6a) and *p*-NPA (Figure 6b). The plot of V_o_ vs. substrate concentration [S] has a classical Michaelis–Menten profile. The kinetic parameters Km and Vmax estimates by non-linear regression are shown. Higher Km and Vmax was calculated for *p*-NPA, (Km = 1.0 mM ± 0.04, Vmax = 0.0011 ± 8.82 × 10^−5^ mM·min·mg); although, the Vmax with *p*-NPL is an order of magnitude lower than for *p*-NPA, it has a higher affinity for the former substrate, so that the catalytic efficiencies with both substrates are in the same order of magnitude. This profile and specific activity values found showed that the enzyme studied it is a bona fide lipase that is active on long-chain insoluble fatty acid esters. As for other lipases, its activity is highly variable and depends on the affinity of the specific substrates.

In the *Litopenaeus vannamei* genome analysis, the authors reported a sequence named an esterase OVCA-like [21], with 100% identity to LvFSH. In this study, we overexpressed, purified, and characterized LvFSH like a lipase enzyme. LvFSH showed high activity using short-chain fatty acid but a higher affinity for long-chain fatty acids, which is a characteristic of lipases.

### 3.9. Immunodetection Assay (Dot Blot) of White Shrimp Protein Lipase (LvFSH)

**Figure 7:**
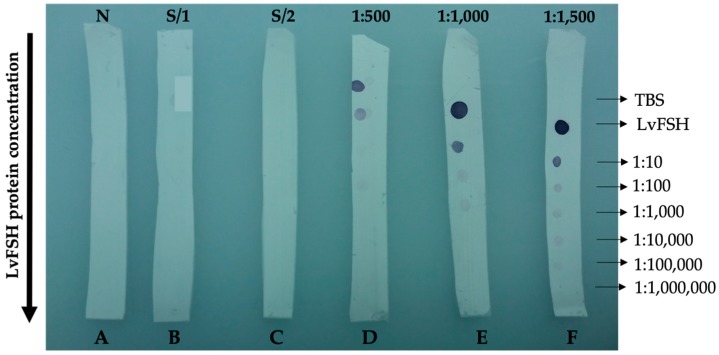
Anti-LvFSH titer by dot-blot assay. A, B, C controls: no antibody (N); B and C: without first (S/1) and second antibody (S/2), respectively; D, E, F, dilution 1:500, 1:1000 and 1:1500 of the obtained anti-LvFSH and purified by protein A chromatography. A total of 2 μL of diluted LvFSH protein solution was applied in descending order: C, 0.228 mg/mL, and a serial dilution (1:10 to 1:1,000,000) and Tris saline buffer (TBS) as control.

Figure 7 shows the results obtained from the dot blot immunodetection test used to obtain the titer of polyclonal anti-LvFSH antibodies generated in a rabbit. As shown in controls A, B, and C (without any antibody, without primary anti-LvFSH antibody, and without the secondary goat anti-rabbit-AP antibody, respectively), we observed no detection, suggesting absence of cross-reactivity and unspecific binding.

The anti-LvFSH polyclonal antibodies produced showed high specificity and efficiency. 1:100,000 dilution of pure protein at an initial concentration of 0.228 mg/L in a 1:1500 dilution of anti-LvFSH was immunodetected.

The first antibody (anti-LvFSH) was used at a ratio of 1:500, 1:1000, and 1:1500 (Figure 7), the most intense signal was obtained in the concentrated protein solution (LvFSH = 228 μg/mL), decreasing the spot intensity as the protein was diluted. The detection range of the 1:500 and 1:1000 anti-LvFSH dilution was up to a 1:1000 dilution of LvFSH, while the 1:1500 antibodies had maximum detection at a 1:100,000 dilution of protein. No detection was observed in controls.

**Figure 8:**
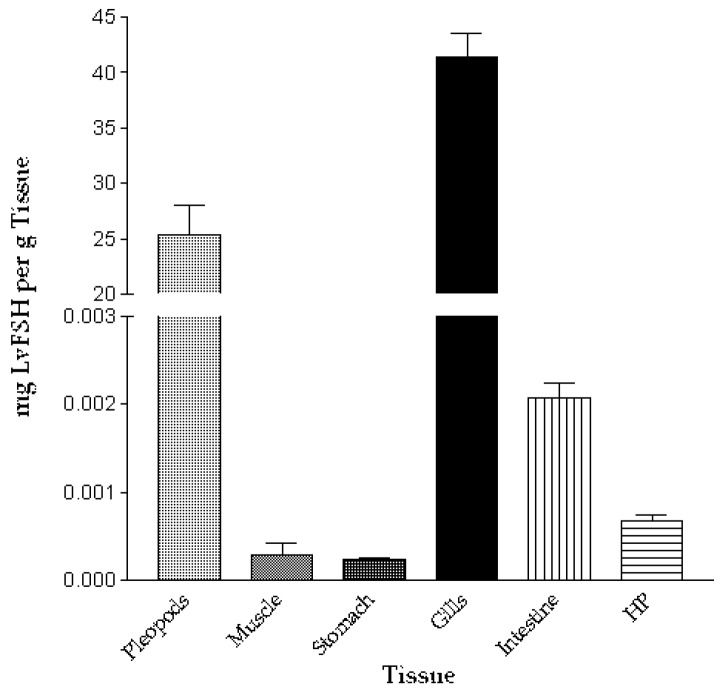
LvFSH immunodetection in *Litopenaeus vannamei* tissues by ELISA.

Before the LvFSH immunodetection in tissue by ELISA, a standard curve was made with pure LvFSH protein. Figure 8 shows the results obtained from the ELISA LvFSH detection in the analyzed tissues. The most substantial amount of LvFSH was in gills and pleopods (41.38 + 4.8 mg/g and 25.40 + 2.6 mg/g tissue, respectively), while the lower level was observed in the stomach (2.30 × 10^−4^ + 4 × 10^−5^ mg/g tissue).

## 4. Discussion

The structure obtained from the modeling of LvFSH carried out in Phyre2 suggests that the protein deduced belongs to the α/β hydrolase family and employs the catalytic triad Ser/Asp/His characteristic of the serine hydrolases enzymes. From the structural analysis, the in vivo biochemical activity of the protein cannot be predicted since there is a wide variety of reactions that are catalyzed by α/β hydrolases enzymes. Despite the above, the hydrophobic nature of the active site of the model obtained suggests that this protein could be involved in the hydrolysis of ester-type lipid compounds. Moreover, the relevant prediction from databases of the probable interaction between LvFSH and the domain LIDH hydrolase PF10230 present in the protein LDH1 (Lipid droplet hydrolase), could be assayed and provide experimental evidence to confirm a role for LvFSH in the mobilization of the stored lipids. In this work, enzymatic activities were carried out with different substrates, in order to test bioinformatic predictions that were made by analyzing the primary and theoretical tertiary structure of LvFSH. Interestingly, the volume of the LvFSH active site is almost identical to the molecular volume of and *p*-NPL. Since the cavity is hydrophobic, the smaller *p*-NPA molecule would leave some ordered water molecules in the site, leading to a higher affinity (smaller Km) for *p*-NPL vs. *p*-NPA.

The polyclonal antibodies should be specific against the antigen for which they were prepared and show selectivity and reproducibility [57,58]. Polyclonal antibodies (pAb), representing a more varied collection of paratopes for the same antigen, provided more probabilities of detection. LvFSH being a novel protein and without knowing its interaction as an antigen, pAb proved to be a good tool for the detection of LvFSH [57] and will enable future immunohistochemical and biochemical inquiries.

An important validation when working with pAbs is cross-reactivity [57,58]. It was found that the anti-LvLIP antibodies did not show cross-reactivity and no detection was shown in the TBS controls.

We used an ELISA assay to quantitate the levels of LvFSH in several shrimp tissues. LvFSH protein was immunodetected in all tissues analyzed except in the posterior intestine. The tissues with higher levels of the LvFSH protein were gills and pleopods (41.38 and 25.40 mg of LvFSH/g tissue, respectively), suggesting that indeed, it is not a digestive enzyme. Rivera and García (2011), identified transcripts of an intracellular and extracellular lipase protein in the digestive glands of shrimp [8]. However, during the fasting periods, the expression of intracellular lipase changed, suggesting lipid mobilization.

A key point to identify the function of a lipase is tissue expression. In general, digestive lipases are only expressed in the gut and intestine, whereas intracellular lipases are not limited to the digestive system. In Rivera et al. (2011), the first shrimp intracellular lipase of 196 kDa was found in pleopods, related to the use of lipid for energy mobilization [35]. The presence of lipases in white shrimp gills has not been reported.

The immunodetection of LvFSH in gills and pleopods may be related to the conditions in which the shrimp used were collected, possibly hypoxic conditions with high suspended organic content. It has been described that during hypoxic conditions where aerobic metabolism decreases, the organism tends to increase the use of its energy reservoir [59,60].

## 5. Conclusions

The bioinformatic analysis led us to identify a novel α/β hydrolase, and the function of the gene product was studied using recombinant expression and a biochemical characterization approach. The *LvFSH* sequence was found in the shrimp *Litopenaeus vannamei* transcriptome, produced as a recombinant protein, and found to have esterase activity and a high affinity (low Km) for a long chain lipid substrate. The amino acid sequence suggests that LvFSH is an intracellular protein and based on molecular and structural similarities (27% identity) with the YHR049W/FSH1 protein [3], this lipase could be part of a eukaryotic family serine hydrolase (FSH).

We propose that the function of LvFSH is more related to signal transduction rather than a digestive function since the amino acid sequence does not contain a signal peptide that would allow secretion to hepatopancreas or intestine. Moreover, the active site volume appears to contain lipids with up to 12 carbon chains, and shrimp metabolizes preferably fats with chains of 14 carbons and longer [61]. Additionally, the multiple protein-protein interactions predicted by bioinformatic methods would be more relevant under the intracellular space with a broader range of interactions, rather than in the digestive lumen where the catalytic function would be more critical. LvFSH is similar to the OVCA2 oncogene, a protein present in tumor proliferation processes [44], and whether that is relevant to crustacean biology, it hints towards lipid mobilization for energy production and to participate in protein-protein interactions. More investigation is mandated to elucidate the function in marine invertebrates.

## Figures and Tables

**Table 1:** Results of Eukaryotic Linear Motif (ELM) motif search within the primary structure of LvFSH.

ELM Name	Instances (Matched Sequences)	Positions	ELM Description
CLV_C14_Caspase3-7	DDKDG	58–62	Caspase-3 and Caspase-7 cleavage site
DOC_MAPK_DCC_7 DOC_MAPK_MEF2A_6	KITLPTLHV	156–164	A kinase docking motif mediating interaction towards the ERK1/2 and p38 subfamilies of MAP kinases
DOC_MAPK_gen_1	RKLLKKQVEF KLLKKQVEF	33–42 34–42	MAPK interacting molecules (e.g., MAPKKs, substrates, phosphatases) carry a docking motif that helps to regulate specific interaction in the MAPK cascade. The classic motif approximates (R/K)xxxx#x# where # is a hydrophobic residue
DOC_PP1_RVXF_1	LKKQVEFE	36–43	Protein phosphatase 1 catalytic subunit (PP1c) interacting motif binds targeting proteins that dock to the substrate for dephosphorylation. The motif defined is [RK]{0,1}[VI][^P][FW]
DOC_PP2B_LxvP_1	LEVP	49–52	Docking motif in calcineurin substrates that binds at the interface of the catalytic CNA and regulatory CNB subunits
DOC_WW_Pin1_4	FMTSPL	44–49	The Class IV WW domain interaction motif is recognized primarily by the Pin1 phosphorylation-dependent prolyl isomerase
LIG_CaM_IQ_9	ICGLQQQGKLSYSFKFA	120–136	Helical peptide motif is responsible for Ca^2+^-independent binding of the CaM. The motif is mainly characterized by a hydrophobic residue at position 1, a highly conserved Gln at position 2, basic charges at positions 6 and 11, and a variable Gly at position 7
LIG_LIR_Gen_1	TGAFRKL	29–35	Canonical LIR motif that binds to Atg8 protein family members to mediate processes involved in autophagy
LIG_MYND_3	VPPLE	51–55	A variant MYND binding motif found in the HSP90 co-chaperones p23 and FKBP38 interacting with PHD2 MYND domain
LIG_PDZ_Class_3	KMRELC	214–219	The C-terminal class 3 PDZ-binding motif is classically represented by a pattern such as (DE)X(VIL)*
LIG_SH2_STAT5	YFHD YVKF	183–186 208–211	STAT5 Src Homology 2 (SH2) domain-binding motif
MOD_CK2_1	NGSTFRE FMTSPLE	21–27 44–50	CK2 phosphorylation site
MOD_NEK2_1	FEESLG LSYSFK	87–92 129–134	NEK2 phosphorylation motif with preferred Phe, Leu, or Met in the −3 position to compensate for less favorable residues in the +1 and +2 position
MOD_Plk_1	FEESLGA	87–93	Ser/Thr residue phosphorylated by the Plk1 kinase
MOD_ProDKin_1	FMTSPLE	44–50	Proline-Directed Kinase (e.g., MAPK) phosphorylation site in higher eukaryotes
TRG_ENDOCYTIC_2	YVKF	208–211	Tyrosine-based sorting signal responsible for the interaction with mu subunit of AP (Adaptor Protein) complex
